# Effect of macular photocoagulation on visual acuity of Omani patients with clinically significant macular edema

**DOI:** 10.4103/0974-620X.53034

**Published:** 2009

**Authors:** Zafar A. Zaidi, Mary K. Jacob

**Affiliations:** Department of Ophthalmology, Nizwa Hospital, Nizwa, Sultanate of Oman

**Keywords:** Diabetic macular edema, diabetic retinopathy, hyperlipedemia, photocoagulation

## Abstract

**Background:**

The aim of this study was to determine the effect of macular laser treatment on the visual acuity (VA) of Omani diabetic patients with clinically significant macular edema (CSME). Visual outcome was also correlated with duration and control of diabetes and presence or absence of hypertension and hyperlipidemia.

**Materials and Methods:**

This is a retrospective noncomparative cohort study involving 101 eyes of 72 Omani diabetic patients. Change in VA was determined using Snellen′s VA chart. The mean duration of follow-up was approximately 21 months (range, 16-24 months).

**Results:**

29.7% of the patients maintained their vision, 35.6% showed improvement, whereas 34.7% showed a decrease in their vision. Positive visual outcome showed a statistically significant direct relationship with tight control of diabetes and absence of hypertension and an inverse relationship with the duration of diabetes. Presence of hyperlipedemia did not show a statistically significant relationship with positive visual outcome. However, it showed a trend to better visual outcome in the absence of hyperlipedemia. Peak incidence of macular edema was seen at the age of 52.3 years.

**Conclusion:**

Macular photocoagulation was found to be an effective method of treatment for CSME among Omani diabetic patients, which has resulted in a positive visual outcome in 65.3% of the patients (stable and improved vision). Effective control of diabetes, duration of diabetes, and hypertension are the factors which influence the postlaser visual outcome.

## Introduction

Diabetic retinopathy is a major cause of blindness in adults from industrialized and developing countries. In spite of patients′ awareness and glycemic control, prevalence of diabetic retinopathy is increasing.[1] Also, in countries such as Oman, where there has been very rapid development, changes in lifestyle have been very significant. Among the estimated 2.4 million Omani population, approximately 10% are being treated for diabetes at any time.[2,3] A hospital-based study of diabetic retinopathy in Oman has shown a prevalence of 14.4%.[2]

Diabetic macular edema (DME) is the most common cause of visual loss among diabetic patients. It can appear at any stage during development of diabetic retinopathy except in early nonproliferative diabetic retinopathy.[1,4] DME is caused by either focal or diffuse leakage from retinal vasculature. Macular photocoagulation is the established mode of treatment for clinically significant macular edema (CSME) as defined by early treatment diabetic retinopathy study (ETDRS) group.[1,5,6]

The most commonly used modes of laser treatment for CSME are: focal, grid, and modified grid. In focal treatment, microaneurysms causing macular edema are treated directly; in grid treatment, the area of diffuse capillary leakage and capillary nonperfusion are lasered in a grid pattern;[6-8] and modified grid is a combination of focal and grid treatments.

The exact action mechanism of laser treatment is yet not explored. It may work through the absorption of laser by melanin pigments in retinal pigment epithelium and choroid, and also by the hemoglobin in the microaneurysm. The increase in oxygen permeability from choroid to inner retina results in decrease of neovascularisation. Also, thrombosis of the microaneurysm decreases the vascular leakage.[9] The treatment may cause initial worsening of VA. It takes 3−4 months to observe the benefit of treatment. Additional treatment should be done in case of persistent macular edema.

In this study we aim to assess the effect of Argon laser treatment on VA of Omani patients with CSME.

## Materials and Methods

This was a retrospective and noncomparative cohort study involving 101 eyes of 72 Omani patients with CSME. In these patients, macular laser treatment was done using Argon Laser in Nizwa Hospital, Oman. Any patient with corneal opacity and lenticular changes that interfered with visualization of the fundus, was excluded from the study.

This study complied with the policies of the institutional Ethical Committee and permission was granted to conduct the study.

The process included review of detailed medical and ocular history and ocular examination. Best corrected visual acuity (BCVA) using Snellen′s visual acuity chart was noted prior to laser photocoagulation and on each follow-up. A detailed anterior segment examination was done using a slit lamp. Posterior segment was examined after maximum pupillary dilatation using tropicamide 1% and phenylephrine 5%. The methods included indirect ophthalmoscopy and biomicroscopy using Volk+90D lens or Goldmann′s 3-mirror contact lens. Indication for treatment was CSME, i.e., retinal thickening that involves or threatens the center of macula (even when vision is not yet affected). All cases of CSME were treated at the earliest according to the recommendations of ETDRS after taking informed written consent. Focal treatment was done for focal maculopathy, grid treatment for diffuse maculopathy, and modified grid treatment for combination of focal and diffuse maculopathy. Treatment was done under topical anaesthesia using Benoxinate hydrocholoride 4% eye drops by a coherent Argon laser machine using green light. Follow-up examination was done after one and three months of laser treatment and subsequently, the frequency of follow-up varied for individual cases depending on the resolution of macular edema.

Retreatment for persistent or new macular edema was done when indicated. Patients were followed up for a mean period of 21 months (range, 16−24 months) after laser treatment.

Data regarding the type, duration, and mode of diabetes treatment was noted. Also, association of hypertension and hyperlipedemia, baseline and regular blood sugar, and blood pressure examination, were noted. Criteria for diabetic control was taken as fasting blood glucose <7 mmol/L on two consecutive visits. Criteria for the presence of hypertension was taken as already diagnosed cases on treatment or blood pressure of >140/90 mm Hg. Whereas, criteria for the presence of hyperlipedemia was taken as already diagnosed cases on treatment or total serum cholesterol >5.2 mmol/dl.

Changes in visual acuity post laser treatment were recorded. These were analyzed with reference to the following: duration of diabetes, control of diabetes, hypertension, and hyperlipedemia. Post laser visual acuity was taken as the BCVA using Snellen′s VA chart during the last follow-up. Post laser stable vision or improvement by at least one line was taken as a positive visual outcome. Significant visual loss was taken as decrease vision of more than two lines of Snellen′s VA chart.

Statistical analysis was done using SPSS-10 package. Chi-square test was used and *P* value of ≤0.05 was considered significantly. For analysis of statistical significance "unknown" was excluded.

## Results

The demographic profile of the patients is shown in Table 1. Majority of patients were in the age group of 50−59 years. The age of patients ranged from 36−71 years with the average being 56.5 years.

**Table 1:** Age and sex distribution

*Age gropus (years)*	*Male*	*Female*	*Total*
Less than 40	2	2	4(4%)
40–49	17	10	27
50–59	28	15	43(42.6%)
60–69	15	8	23
70 & more	2	2	4(4%)
Total	64	37	101
	(63.4%)	(36.6%)	(100%)

Majority of the eyes (81.2%, n = 82) had an associated moderate nonproliferative diabetic retinopathy. Severe nonproliferative diabetic retinopathy was seen in 16.8% (n = 17) and proliferative diabetic retinopathy in 2% (n = 2).

The duration of follow-up ranged from 16−24 months.

Considering the visual outcome as a whole: 29.7% of the patients maintained their VA; 35.6% showed improvement in vision; and 34.7% showed a decrease in VA. Visual improvement in terms of VA was as follows: 27.6% improved by up to one line of Snellen′s VA chart, 7% by one to two lines, and 1% by more than two lines. Also, 34.7% patients showed a decrease in the VA; among them, 23.8% had a decrease by one line, 9.9% by one to two lines, and 1% by more than two lines. Significant visual loss (decrease in VA of two or more lines) was observed in 4% [Table 2].

**Table 2:** Correlation of visual outcome to prelaser visual acuity

*Pre laser Vision*	*Number of eyes with post laser visual improvement*							
	*>2 Lines*	*Upto 2 lines*	*Upto 1 line*	*Stable*	*Dec 1 line*	*Dec 2 lines*	*Dec >2 lines*	*Total*
6/6.6/6p	0	0	3	6	2	0	0	11
6/9p-6/9	0	0	11	5	3	5	1	25
6/12p-6/12	0	2	4	8	12	5	0	31
6/18p-6/18	0	0	4	6	3	0	0	13
6/24p-6/24	1	1	4	2	3	0	0	11
6/36p-6/36	0	0	1	2	0	0	0	3
6/60 and below	0	4	1	1	1	0	0	7
Total	1	7	28	30	24	10	1	101

**Figure 1:**
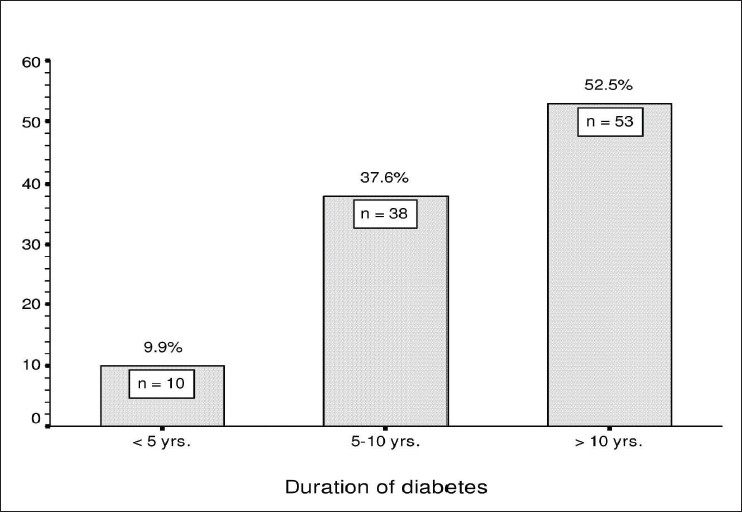
Prevalence of macular edema with respect to age

Prevalence of macular edema with respect to age [Figure 1] was seen varying from 30−80 years with the peak incidence at 52.3 years.

**Figure 2:**
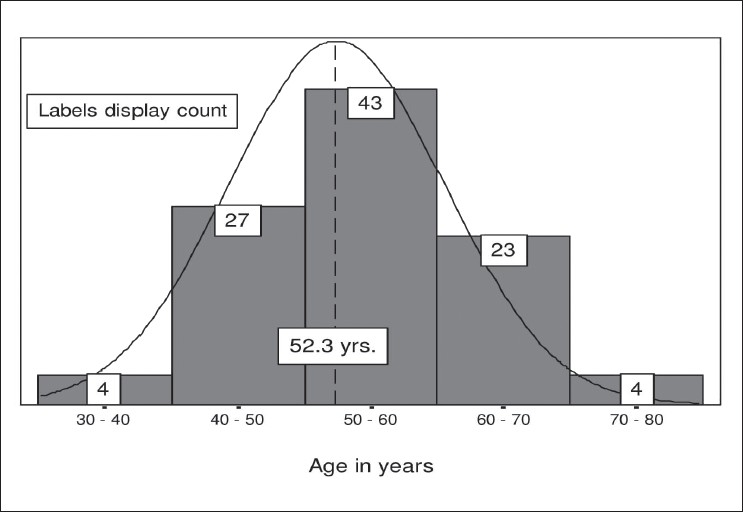
Relation of CSME with duration of diabetes

Figure 2 shows the relationship of CSME in our cases with duration of diabetes.

**Figure 3:**
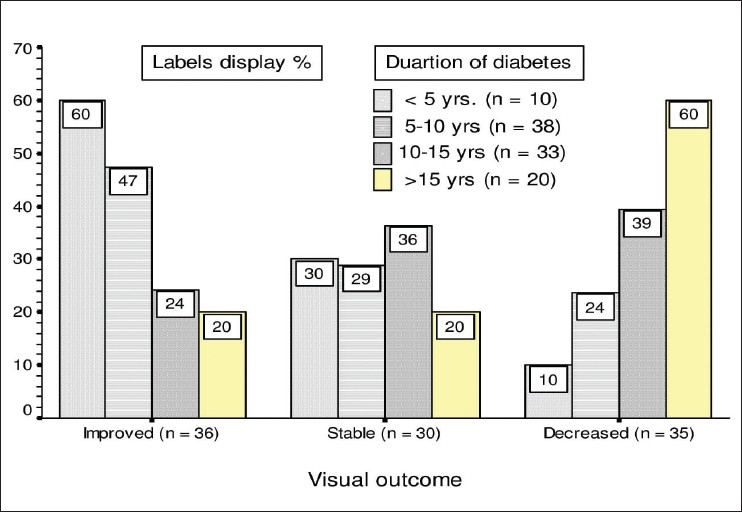
Effect of duration of diabetes on visual outcome

The duration of diabetes at presentation and its relationship with the post laser visual outcome is shown in Figure 3 and Table 3. These show an inverse relationship between the duration of diabetes and a positive visual outcome post laser. The longer the duration of diabetes mellitus, the poorer the post laser visual outcome. This relationship is statistically significant (*P* = 0.013).

**Table 3:** Correlation of visual outcome to duration of diabetes

*Visual outcome*	*Control of Diabetes (years)*				*Subtotal*	*Total*
	*<5*	*5-10*	*10-15*	*>15*		
Improved	6	18	8	4	36	66
	(60%)	(47.4%)	(24.2%)	(20%)		
Stable	3	11	12	4	30	
	(30%)	(28.9%)	(36.4%)	(20%)		
Decreased	1	9	13	12	35	35
	(10%)	(23.6%)	(39.4%)	(60%)		
Total	10	38	33	20	101	101

Figures in parentheses are in percentage

Table 4 shows the correlation of the control of diabetes with the visual outcome. Patients who had well-controlled diabetes have had a statistically significant positive visual outcome (*P* = 0.008).

**Table 4:** Visual outcome in relation to control of Diabetes

*Visual outcome*	*Control of Diabetes*		*Subtotal*	*Total*
	*Controlled*	*Uncontrolled*		
Improved	19	17	36	
	(46.3)	(28.3)	(35.7)	66
Stable	14	16	30	
	(34.1)	(26.7)	(29.7)	
Decreased	8	27	35	
	(19.5)	(44.9)	(34.7)	35
Total	41	60	101	101

Figures in parentheses are in percentage

Table 5 shows the correlation of visual outcome with hypertensive status (12 patients of unknown hypertensive status were excluded from statistical analysis). Absence of hypertension has been seen to be associated with a positive visual outcome. This association is statistically significant (*P* = 0.022).

**Table 5:** Correlation of visual outcome with hypertension

*Visual outcome*	*Hypertension*			*Subtotal*	*Total*
	*Normotensive*	*Hypertensive*	*Unknown*		
Improved	13	22	1	36	66
	(43.3)	(37)	(8.3)		
Stable	12	13	5	30	
	(40)	(22)	(41.7)		
Decreased	5	24	6	35	35
	(16.7)	(40.7)	(49.9)		
Total	30	59	12	101	101

Figures in parentheses are in percentage

Our study does not reveal any statistically significant relationship between the presence of hyperlipidemia and visual outcome (*P* = 0.182) [Table 6].

**Table 6:** Correlation of visual outcome with Hypercholesterolemia

*Visual outcome*	*Hypercholesterolemia*			*Subtotal*
	*Not present*	*Present*	*Unknown*	
Imptoved	11	16	9	
	(32.3)	(39)	(34.5)	36
Stable	15	7	8	
	(44.1)	(17.1)	(30.8)	30
Decreased	8	18	9	
	(23.5)	(43.9)	(24.6)	35
Total	34	41	26	101

Figures in parentheses are in percentage

## Discussion

In patients with diabetic retinopathy, laser treatment is directed at prevention of visual loss rather than visual improvement. Laser treatment usually does not improve vision once it has decreased.[7,10] It should be offered before the occurrence of visual loss, when the risk of visual loss justifies the adverse effect of laser treatment.[11] In our study, we have included all cases with stable or one line improvement in VA as positive outcome.

In this study, we followed the study definitions of ETDRS with respect to diagnosis and treatment of CSME.[7,8] Early efforts at treating macular edema with laser before significant visual loss occurs yielded favorable results. The literature review of similar photocoagulation studies in DME such as Olk *et al*.,[12,13] the British multicenter study group,[14] Blankenship[15] and ETDRS research group,[5] has shown that macular photocoagulation is effective in treating macular edema and in stabilizing vision.[7] ETDRS showed that photocoagulation decreased persistent macular edema and significant visual loss by 50%.[5] A recent systematic review of well-conducted studies related to management of diabetic retinopathy had showed that focal laser treatment reduced the risk of moderate visual loss in 50−70% of patients with DME.[16]

Final visual outcome in our study group showed that 29.7% of patients maintained their baseline VA and 35.6% of patients showed improvement in vision. This accounts for an overall positive effect on VA in 65.3% of the treated patients.

Olk *et al*., showed that macular laser treatment resulted in visual improvement in 33% of cases taking the criteria for visual improvement as at least two lines of Snellen′s VA chart.[7] visual improvement criteria in our study was taken as at least one line. After Implementation of this criteria, visual improvement was noted in 29.8% of cases. 15.9% of cases showed two lines of improvement. This difference in efficacy may be related to the difference in the degree of visual impairment at the start of the treatment. However, due to lack of comparable data the exact reason could not be analyzed. United Kingdom Prospective Diabetes Study (UKPDS) among the 40.5% of patients who showed decrease in VA, 3% had significant visual loss in spite of laser treatment i.e. loss of more than two lines of Snellen′s VA chart. This is in contrast to the Olk *et al*., where the significant visual loss was 4% following similar study definitions.[7,12,13] In ETDRS, significant visual loss occurred in 5% and 7% of treated eyes after one and two years, respectively,[5,7] where the criteria for significant visual loss was taken as more than three lines.

Peak incidence of macular edema in our study was found to be at 52.3 years, which correlated with the observation in Wisconsin epidemiological study of diabetic retinopathy where the peak incidence of macular edema was at 51 years.[7,17]

Retinal arteriolar hemodynamic was found to be positively related to age, duration of diabetes, and hypertension.[1] Numerous clinical trials have proved that the complications of diabetes including those related to eyes can be decreased by good control of diabetes and hypertension.[9] All complications arising from diabetes are due to hyperglycemia; and chronic hyperglycemia affects the blood vessels leading to vascular dysfunction.[9,18]

Glycemic control is an identified risk factor for retinopathy progression as well as DME as evidenced by diabetes control and complication trial (DCCT). In the follow up study of this cohort, the Epidemiology of Diabetes Intervention and Control (EDIC) study demonstrated that intensive therapy had a lower rate of progression to DME requiring laser treatment than those in the good control group.[19] In our study, we have found an inverse relationship between duration of diabetes and positive visual outcome after laser treatment, which is statistically significant. This study also revealed a positive postlaser visual outcome in relation to control of diabetes.

Incidence of hypertension is more in diabetic patients and thought to be a risk factor in the development of diabetic retinopathy.[5,15,20] Approximately 60% of adults with Type 2 diabetes have comorbid hypertension.[21] A follow-up study of hypertensive diabetics showed that tight control of hypertension decreased the risk of diabetic complications compared to less aggressive control.[16,22]

Prospective Diabetes Study (UKPDS), the largest clinical research study of diabetes ever conducted also supports this fact.[23] Our study has shown a statistically significant relationship between absence of hypertension and positive visual outcome. However a recent study conducted in Oman by Keshav *et al*, did not show a direct correlation between visual outcome with respect to control of diabetes and presence of hypertension.[24] This may be attributed to the short duration of follow up.

Hyperlipidemia is also found to be a risk factor for diabetic retinopathy. It has been postulated that excessive dietary exposure to fats may contribute to macular edema perhaps through lipid peroxidation or other vasculopathic influences.[25] Treatment of hyperlipidemia provides cardiovascular benefits[26,27] and has shown to be effective as an adjunct in the management of CSME.[28] Observational data from the ETDRS suggests that lipid lowering may decrease the risk of exudate formation and hence visual loss in patients with diabetic retinopathy.[26] Another prospective study concluded that serum lipid fraction may affect the success of laser treatment and also the course of macular edema.[29] Our study failed to show any statistically significant relationship between presence or absence of hypercholesterolemia with a positive visual outcome. However, there appears to be a trend towards better visual improvement in patients without hyperlipidemia.

We do acknowledge the limitation of this study due to lack of facilities. Monitoring and quantifying macular edema using optical coherence tomography (OCT) would have been a more objective predictor along with the VA in assessing the effect of laser treatment. Also glycosylated hemoglobin (HBA1C) measurement would have been a more accurate indicator of glycemic control. Similarly, lipid fraction was not assessed in this cohort and as discussed above it is considered to be superior than total cholesterol evaluation.

New understanding of the underlying pathophysiology of DME has provoked interest in alternative modes of treatments such as intraocular steroids, antivascular endothelial growth factor, and protein kinase c-beta inhibition. However, a recent study comparing long-term visual outcome, following intravitreal steroids and photocoagulation, showed superiority of photocoagulation over the former.[30] Laser photocoagulation according to guidelines of ETDRS continues to be the primary modality of treatment. Also, excellent metabolic management and control of comorbidities should be the main objective at any phase of the disease.
